# HMGB1 and Caveolin-1 related to RPE cell senescence in age-related macular degeneration

**DOI:** 10.18632/aging.102039

**Published:** 2019-07-07

**Authors:** Shuo Sun, Bincui Cai, Yao Li, Wenqi Su, Xuzheng Zhao, Boteng Gong, Zhiqing Li, Xiaomin Zhang, Yalin Wu, Chao Chen, Stephen H. Tsang, Jin Yang, Xiaorong Li

**Affiliations:** 1 Tianjin Key Laboratory of Retinal Functions and Diseases, Eye Institute and School of Optometry, Tianjin Medical University Eye Hospital, Tianjin, People's Republic of China; 2 Edward S. Harkness Eye Institute, New York-Presbyterian Hospital, New York, NY 10032, USA; 3 Departments of Ophthalmology, Columbia University, New York, NY 10027, USA; 4 Tangshan Eye Hospital, Tangshan, People's Republic of China; 5 Fujian Provincial Key Laboratory of Ophthalmology and Visual Science, Eye Institute of Xiamen University, College of Medicine, Xiamen University, Xiamen City, People's Republic of China

**Keywords:** A2E, HMGB1, Caveolin-1, RPE cell senescence, AMD

## Abstract

Accumulation of lipofuscin in the retinal pigment epithelium (RPE) is considered a major cause of RPE dysfunction and senescence in age-related macular degeneration (AMD), and *N*-retinylidene-*N*-retinylethanolamine (A2E) is the main fluorophore identified in lipofuscin from aged human eyes. Here, human-induced pluripotent stem cell (iPSC)-RPE was generated from healthy individuals to reveal proteomic changes associated with A2E-related RPE cell senescence. A novel RPE cell senescence-related protein, high-mobility group box 1 (HMGB1), was identified based on proteomic mass spectrometry measurements on iPSC-RPE with A2E treatment. Furthermore, HMGB1 upregulated Caveolin-1, which also was related RPE cell senescence. To investigate whether changes in HMGB1 and Caveolin-1 expression under A2E exposure contribute to RPE cell senescence, human ARPE-19 cells were stimulated with A2E; expression of HMGB1, Caveolin-1, tight junction proteins and senescent phenotypes were verified. HMGB1 inhibition alleviated A2E induced cell senescence. Migration of RPE cells was evaluated. Notably, A2E less than or equal to 10μM induced both HMGB1 and Caveolin-1 protein upregulation and HMGB1 translocation, while Caveolin-1 expression was downregulated when there was more than 10μM A2E. Our data indicate that A2E-induced upregulation of HMGB1、Caveolin-1 and HMGB1 release may relate to RPE cell senescence and play a role in the pathogenesis of AMD.

## Introduction

Age-related macular degeneration (AMD) is the leading cause of vision loss in older adults worldwide [1]. AMD can be classified into early-stage or late-stage AMD. The latter is characterized by neovascularization (wet AMD), geographic atrophy (dry AMD), or both [2]. Conversely, early-stage AMD is characterized by a limited amount of drusen, which is mainly caused by lipid and protein accumulation and thought to contribute to atrophic changes. As the disease progresses, neovascular changes or geographic atrophy involving the macular area can be present in patients for years. Dry AMD manifests as well-demarcated areas, providing direct visualization of the underlying choroidal vessels due to atrophy of photoreceptor and retinal pigment epithelium (RPE) cells; wet AMD is characterized by the development of choroidal neovascularization (CNV) [3]. Although anti-vascular endothelial growth factor (anti-VEGF) has become the main treatment approach for wet AMD, there is a lack of consensus regarding the treatment of dry AMD. Most importantly, an appropriate disease model that can simulate the occurrence and development of AMD must be chosen [4]. Therefore, we explored the relationship between dry AMD and RPE dysfunction and senescence using proteomic mass spectrometry to examine differential expression in induced pluripotent stem cell(iPSC)-derived RPE cell lines with and without A2E treatment [5]. We have previously demonstrated that the iPSC-derived RPE is phenotypically and functionally similar to the native RPE [6]. In addition, the young status of iPSC-RPE may provide an excellent means for observing changes in protein expression during the process of RPE cell aging [7].

As a by-product of the visual cycle, *N*-retinylidene-*N*-retinylethanolamine (A2E) and its isomers are formed by the reaction of two trans-retinal molecules with phosphatidyl-ethanolamine. A2E is the major fluorophore identified in lipofuscin from aged human eyes, and it has been widely studied [8]. As a hallmark of aging, A2E continuously accumulates in the RPE [9]. The lipofuscin constituents consist of various molecules that have photoreactive properties and undergo photo-oxidation [10]. A2E photo-oxidation products can cause oxidative stress, membrane permeation, telomere dysfunction and accelerated RPE senescence [11]. Although A2E is clearly present in the retina, there are rather different opinions regarding its distribution. Ablonczy et al. showed that levels of A2E decreased from the periphery to the centre region in aging tissue of macaques and humans [12] but A2E was localized mainly in the centre region of young mouse retina. However, the distribution of A2E increases across the entire RPE with age [13]. Thus, the relationship between A2E and AMD is worthy of further study.

Our aim in the present study was to identify protein changes related to A2E in aging iPSC-RPE cells and to verify and explore the mechanism of these altered proteins in human ARPE-19 cells.

## RESULTS

### Proteomic mass spectrometry detection of differential expression of proteins, highlighting HMGB1 in iPSC-RPE cells with and without A2E treatment

**Figure 1:**
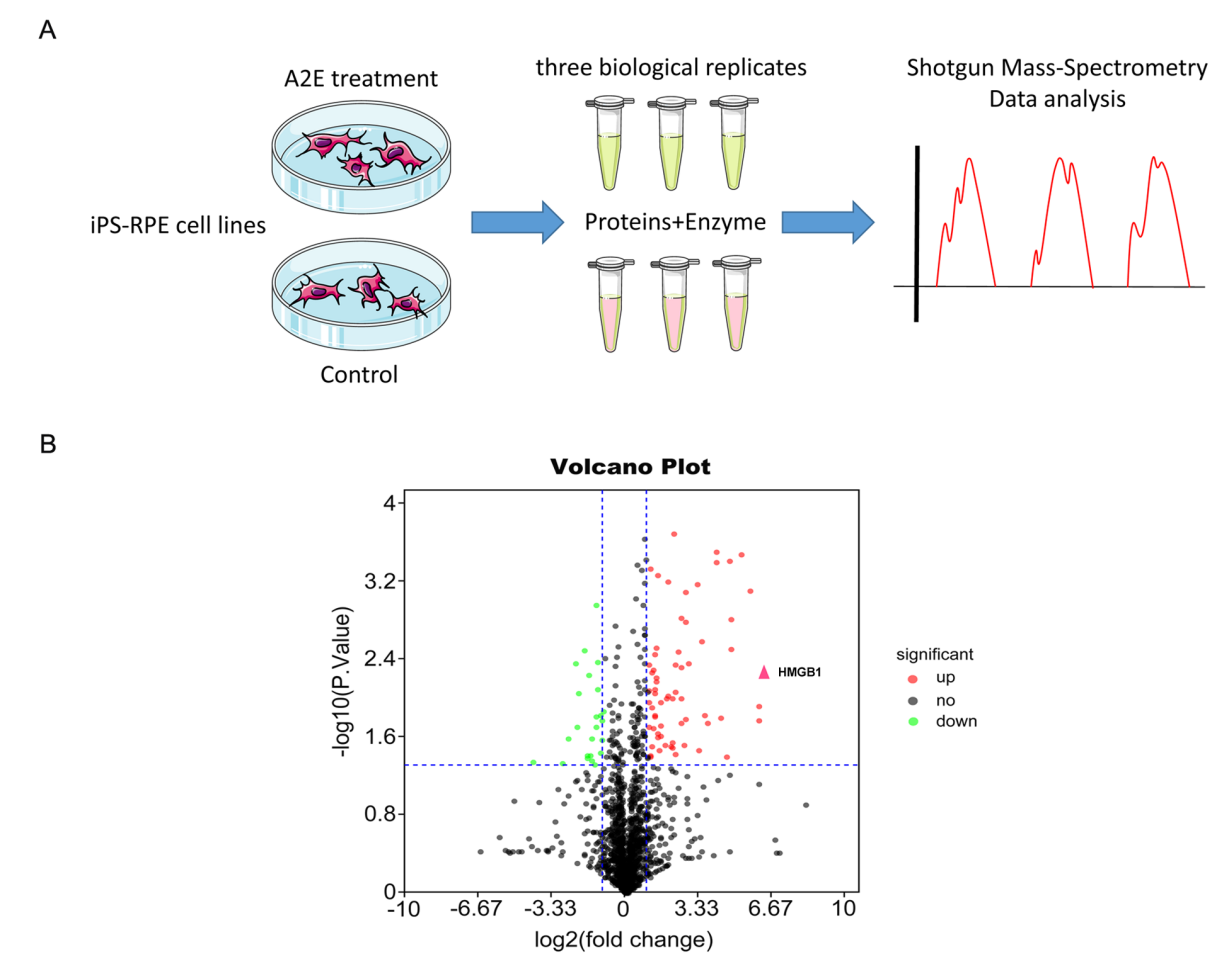
**Proteomic mass spectrometry-based measurement of differential expression of HMGB1.** (**A**) The flow chart of shotgun mass spectrometry. (**B**) Volcano plot illustrating significant differential abundant proteins based on quantitative analysis. The -log_10_ (P value) was plotted against log_2_(fold change A2E treatment/Control). Proteins were significantly upregulated (red dots) or downregulated (green dots) between the A2E treatment and control. The red arrowhead indicates HMGB1.

We used proteomic mass spectrometry to explore differential expression of proteins in iPSC-RPE cells after A2E treatment. The method of iPSC-RPE cell culture was described previously [6]. For liquid chromatography with tandem mass spectrometry (LC-MS/MS) analysis, we extracted proteins from iPSC-derived RPE cells with and without A2E treatment, with three biological replicates prepared from three separate cultures (Flow chart, Figure 1A). Representative proteomic MS-based analyses of proteins from A2E-treated cells versus untreated cells are depicted in a volcano plot in Figure 1B, where the -log_10_(P value) was plotted against the log_2_(fold change A2E Treatment/Control). In the figure, black, green, and red splashes indicate proteins without significant differential expression, significantly downregulated proteins, and significantly upregulated proteins, respectively. We arranged the ratio of A2E treatment/control expression from large to small and found that the high-mobility group box 1(HMGB1), which is marked with a red arrowhead, was upregulated 76-fold in the A2E treatment group compared to the control (*p* value=0.00578,Table 1). Thus, based on MS results, HMGB1 was upregulated in iPSC-RPE cells by A2E treatment.

**Table 1:** The data set was arranged by the ratio of A2E treatment/control, and HMGB1 was found to be upregulated more than 76-fold in the A2E-treated group.

Uniprot#	Gene Names	Ratio A2E treatment/ control	Value A2E treatment/control
P09429	HMGB1	76.95828543	0.0057787
Q06210	GFPT1	65.83452999	0.0167382
G5E9P1	ITPR1	64.99257944	0.0757587
Q7Z4H8	KDELC2	64.17736137	0.0116703
P20591	MX1	50.71424385	0.000773586
P04179	SOD2	37.3876135	0.000328524
H9KVA0	TYMP	27.61593536	0.00153441
F5H090	UNC13C	26.74494289	0.00305275
H3BPK7	AARS	26.39582865	0.000375675
P51911	CNN1	26.24793326	0.0604881
Q8IYM0	FAM186B	25.5978875	0.373977
P21281	ATP6V1B2	23.45846962	0.0387506
Q562R1	ACTBL2	19.38697164	0.0155401
P42785	PRCP PCP	17.65006048	0.0672487
Q15349	RPS6KA2	17.34576845	0.000390284
H0Y9R5	SNX25	17.23254402	0.000304062
Q14240	EIF4A2	15.7087038	0.402011
J3KSW2	POLI	13.22929896	0.0174629
E7EX17	EIF4B	12.53696128	0.106437
P62310	LSM3	11.81044422	0.0147819
Q03135	CAV1	0.810928595	0.289982
P42224	STAT1	4.42761394	0.000198283
P05362	ICAM1	2.45723684	0.00874002

### Upregulation and translocation of HMGB1 in ARPE-19 cells after A2E treatment

**Figure 2:**
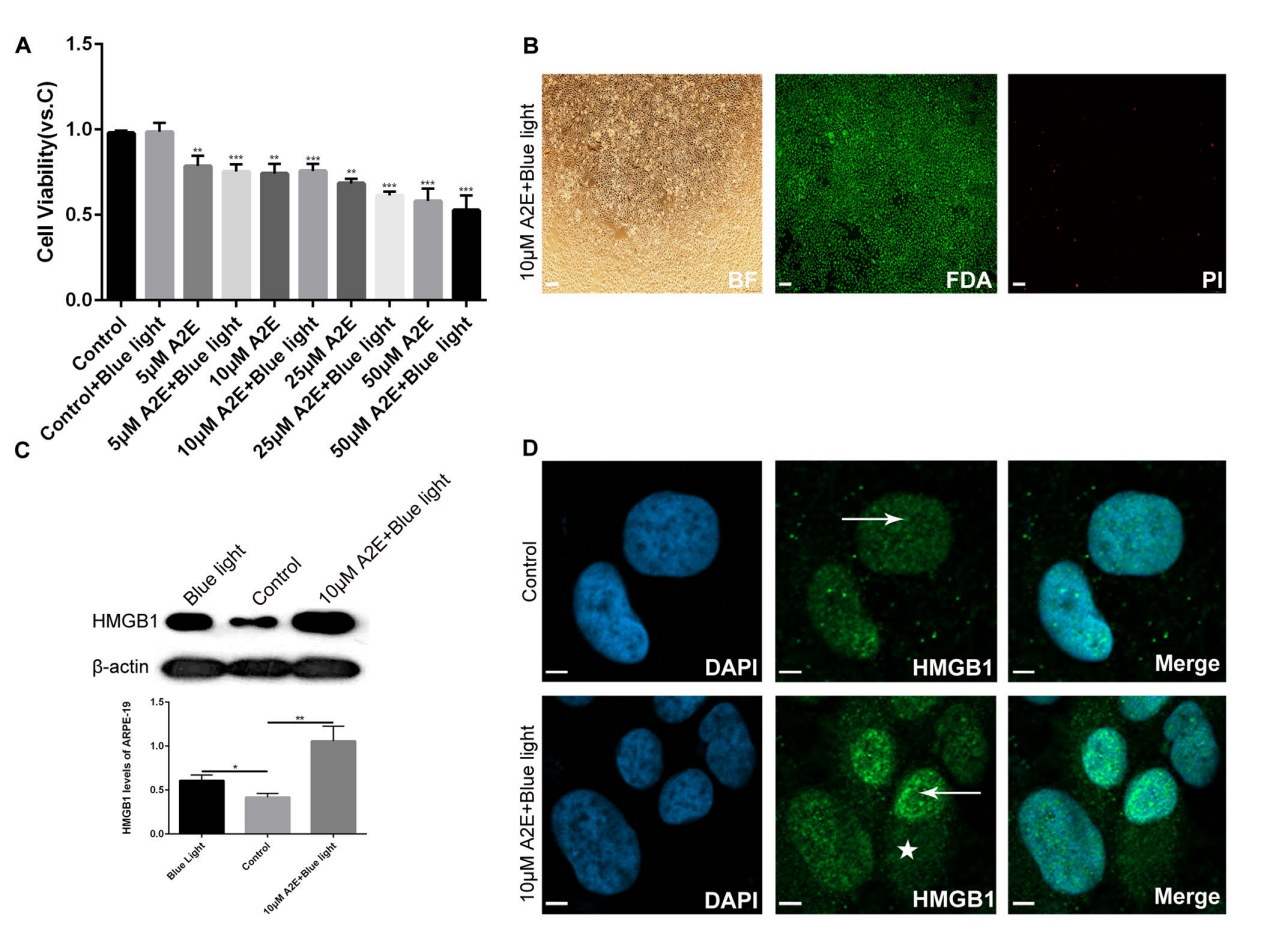
**Experimental validation that blue light exposure of A2E-treated ARPE-19 cells induces HMGB1 upregulation and translocation.** (**A**) An MTT assay was performed on RPE cells treated with different concentrations of A2E with or without blue light photosensitization. Data are presented as means ± SD; * indicates a *p* value < 0.05, ** indicates a *p* value < 0.01, *** indicates a *p* value < 0.001, compared to the control, n=3. (**B**) FDA/PI staining of RPE cells after *in vitro* culture for 48 h with 10 μM A2E + blue light (10 min). Most living RPE cells were stained green by fluorescein diacetate (FDA); a few dead cells were stained red bypropidium iodide (PI). (**C**) Western blot analyses showed that HMGB1 protein expression was higher in 10μM A2E + blue light-treated cells compared to the control and also higher in the blue light treatment, as quantified by densitometry; the results are expressed as a ratio with β-actin. Data are presented as means ± SD; * indicates a *p* value < 0.05, ** indicates a *p* value < 0.01, n=3. (**D**) HMGB1 localization in RPE cells was assessed by confocal microscopy after 10μM A2E + blue light treatment. HMGB1 moved from the nucleus (arrow) to the cytoplasm (star) after 10μM A2E + blue light treatment. Nuclei are labelled with DAPI (blue); HMGB1 is stained green.

To determine the optimized concentration of A2E causing upregulation of HMGB1 without undue influence on cell viability, ARPE-19 cells were incubated with increasing concentrations of A2E with or without blue light (10min) for 48 h. After 24 h in fresh medium, cell viability was examined using the 3-(4, 5-dimethylthiazol-2-yl)-2,5-diphenyl tetrazolium bromide (MTT) assay. The viability of ARPE-19 cells decreased with increasing A2E concentration, especially at 25 μM、 and 50 μM A2E with blue light (Figure 2A). Therefore, we used A2E at a concentration of 10 μM in this study to mimic aged ARPE-19 cells with lipofuscin accumulation. Western blotting of cells incubated with 10 μM A2E with blue light for 48 h showed higher levels of HMGB1 than that of the control and blue light alone (Figure 2C). Moreover, fluorescein diacetate (FDA)/propidium iodide (PI) staining showed that most of the ARPE-19 cells were alive (Figure 2B), confirming that A2E can increase expression of HMGB1 at an early stage and low dose (* indicates a *p* value < 0.05, ** indicates a *p* value < 0.01, *** indicates a *p* value < 0.001). In the presence of A2E, a large amount of HMGB1 was translocated from the nucleus to the cytoplasm (Figure 2D). The results confirm that A2E can induce upregulation and translocation of HMGB1.

### HMGB1 upregulation and release increased the expression of Caveolin-1

**Figure 3:**
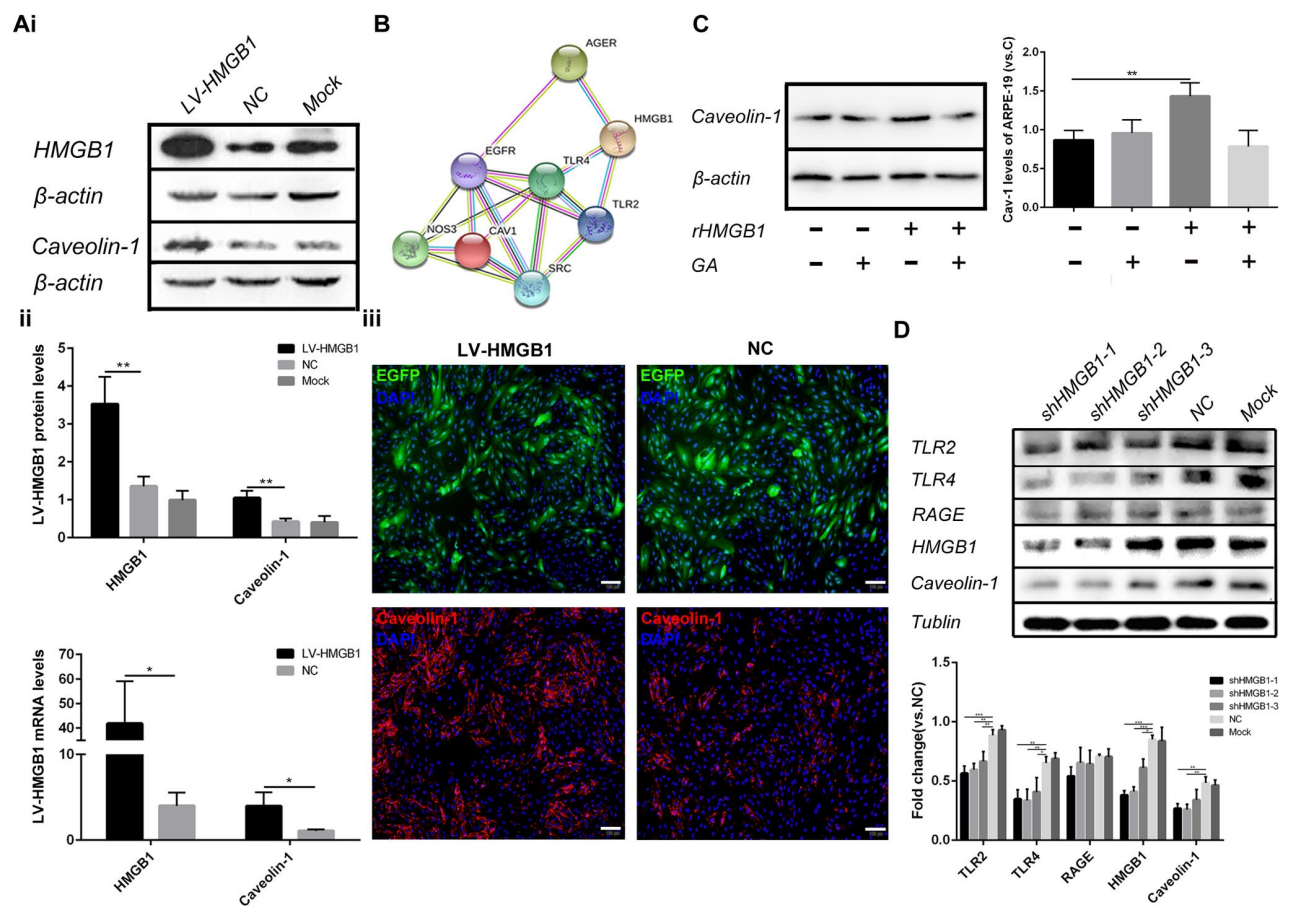
**HMGB1 upregulation and release increase the expression of Caveolin-1.** (**A**) (i) Western blot analyses showed that overexpression of HMGB1 upregulated Caveolin-1; β-actin was used as the loading control; Western blot results were quantified by densitometry, and the results are expressed as a ratio with β-actin. (ii) qPCR analyses showed that overexpression of HMGB1 upregulated Caveolin-1. Data are presented as means ± SD; * indicates a *p* value < 0.05, ** indicates a *p* value < 0.01, n=3. (iii) Expression of EGFP and Caveolin-1 was assessed by immunoﬂuorescence in HMGB1-overexpressing RPE cells and negative-control RPE cells. (**B**) Protein interaction between HMGB1 and Caveolin-1 was revealed by the STRING version 9.1 program. (**C**) Relative Caveolin-1expression in RPE cell incubated with normal medium, 1μg/ml rHMGB1, 100μM GA, or 1μg/ml rHMGB1+100μM GA, Data are presented as means ± SD; * indicates a *p* value < 0.05, ** indicates a *p* value < 0.01, n=3. (**D**) Western blot analyses showed that knock-down of HMGB1 downregulated Caveolin-1; Tublin was used as the loading control, western blot results were quantified by densitometry, and the results are expressed as a ratio with Tublin. Data are presented as means ± SD; * indicates a *p* value < 0.05, ** indicates a *p* value < 0.01, n=3.

The potential role of HMGB1 upregulation and translocation in ARPE-19 cells was then investigated. Cell senescence can be caused by various factors, including DNA damage and oxidative stress. It has been reported that Caveolin-1 plays a major role in cell senescence and that HMGB1 increases its expression [14,15]. The interaction between HMGB1 with Caveolin-1 was assessed using the Search Tool for the Retrieval of Interacting Genes/Proteins (STRING) database (Figure 3B). Thus, RPE cells were infected with HMGB1 overexpression lentivirus、 LV-empty-vector(NC) and stimulated with recombination HMGB1. Then, Real-time Quantitative polymerase chain reaction(qPCR), western blot and immunoﬂuorescence analyses indicated that Caveolin-1 expression was increased by HMGB1 in ARPE-19 cells (Figure 3A,C). Furthermore, lentiviral infection of ARPE-19 cells using shHMGB1 and sh-NC (scramble shRNA) constructs was performed. Effective knock-down of HMGB1 and decrease of Caveolin-1 in ARPE-19 cells transfected with shHMGB1 was demonstrated. Meanwhile, shHMGB1-expressing cells indicated a significant reduction in Toll-like receptor2 (TLR2) and Toll-like receptor4 (TLR4) protein expression but not in Receptor of Advanced Glycation Endproducts (RAGE) which three proteins were reported as potential connection with HMGB1 and Caveolin-1 compared to sh-NC (scramble shRNA) cells. (Figure 3D, * indicates a *p* value < 0.05, ** indicates a *p* value < 0.01, *** indicates a *p* value < 0.001). Together, these results showed that HMGB1 regulates the expression of Caveolin-1 via TLR2 and TLR4.

### Caveolin-1 upregulation induced ARPE-19 cell senescence

**Figure 4:**
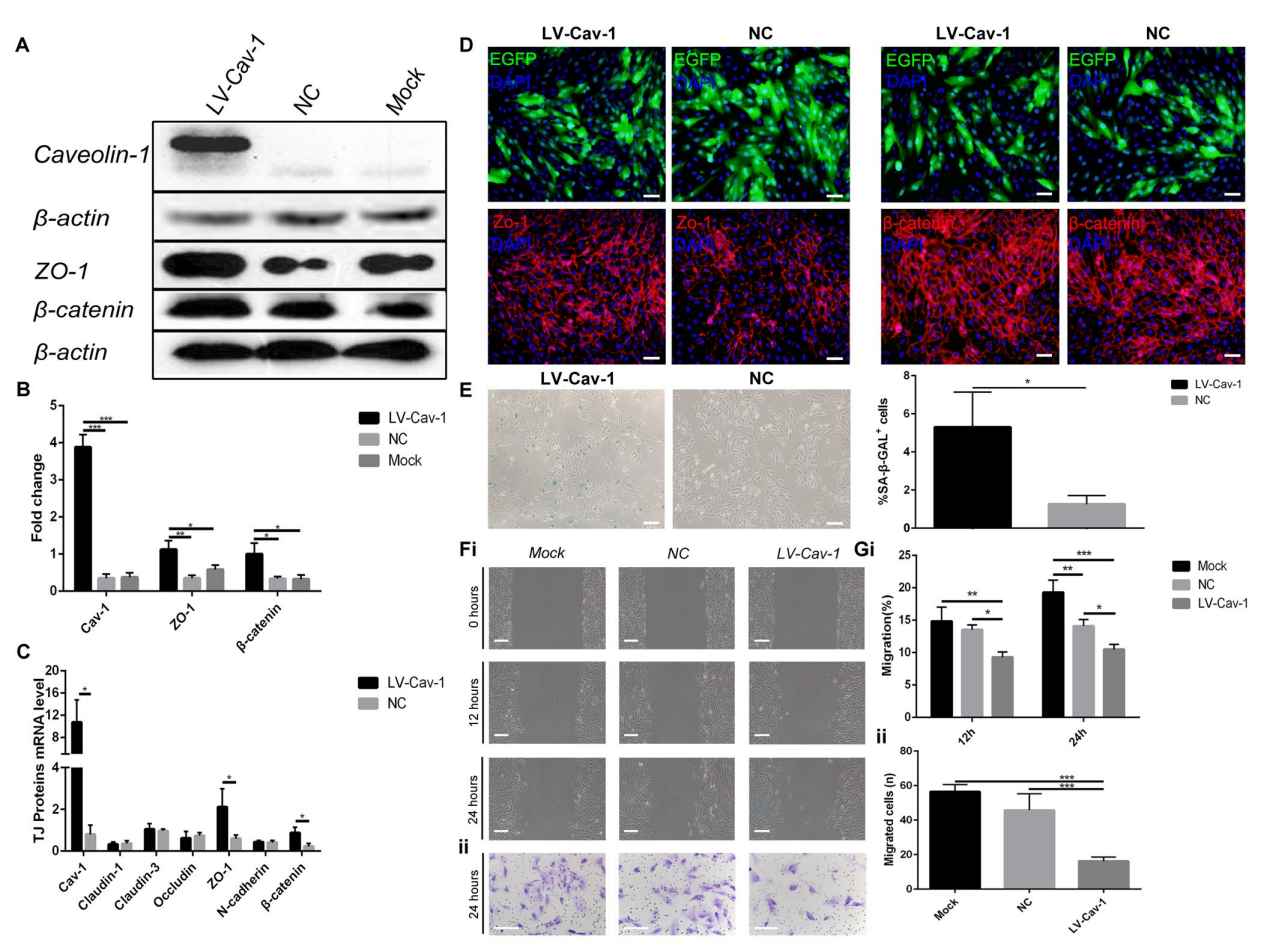
**Overexpression of Caveolin-1 induced ARPE-19 cell senescence and inhibited migration and invasion.** (**A**) Western blot analyses showed that overexpression of Caveolin-1 upregulated Zo-1 and β-catenin; β-actin was used as the loading control. (**B**) Western blot results were quantified by densitometry, and the results are expressed as a ratio with β-actin. Data are presented as means ± SD; * indicates a *p* value < 0.05, ** indicates a *p* value < 0.01, *** indicates a *p* value < 0.001, n=3. (**C**) qPCR analyses showed that overexpression of Caveolin-1 upregulated Zo-1 and β-catenin. Data are presented as means ± SD; * indicates a *p* value < 0.05, n=3. (**D**) Expression of EGFP, Zo-1 and β-catenin was assessed by immunoﬂuorescence in Caveolin-1-overexpressing RPE cells and negative-control RPE cells. (**E**) Representative microscopic images of β-galactosidase staining in RPE cells showed overexpression of Caveolin-1 in RPE cells compared with that in negative-control RPE cells. Quantification of percentage of cells with positive SA-β-gal staining.Data are presented as means ± SD; * indicates a *p* value < 0.05, ** indicates a *p* value < 0.01, n=3. (**F**) (i) Wound-healing assays in Caveolin-1-overexpressing RPE cells. (ii). Transwell invasion assays in Caveolin-1-overexpressing RPE cells. (**G**) (i) The rate of cell migration in different groups was measured at different time points. Note that cell migration was decreased in Caveolin-1-overexpressing RPE cells. (ii) The mean number of invaded cells was assessed in 5 fields. Note that cell invasion was decreased in Caveolin-1-overexpressing RPE cells. Data are presented as means ± SD; * indicates a *p* value < 0.05, ** indicates a *p* value < 0.01, *** indicates a *p* value < 0.001, n=3.

We investigated the effect of stable Caveolin-1 overexpression on ARPE-19 cell senescence. ARPE-19 cells were infected with lentivirus-Caveolin-1, and β-galactosidase staining showed that Caveolin-1-overexpressing RPE cells were more aged compared with the negative control (LV-empty-vector) RPE cells (Figure 4E).

### Inhibition of cell motility by Caveolin-1 upregulation in ARPE-19 cells

Since cell senescence may result in reduced migration and invasion, we further investigated whether Caveolin-1 affects RPE cell migration and invasion capacities using wound-healing and Transwell invasion assays. The results showed that Caveolin-1 overexpression significantly reduced migration (Figure 4Fi and Gi) and invasion (Figure 4Fii and Gii). In addition, expression of Zo-1 and β-catenin was increased by Caveolin-1 upregulation, according to quantitative real-time PCR, western blot and immunoﬂuorescence analysis (Figure 4A, B, C, D). In contrast, the mRNA levels of other tight junction proteins, such as Claudin-1, Claudin-3, Occludin, and N-cadherin, did not change (Figure 4C,* indicates a *p* value < 0.05, ** indicates a *p* value < 0.01, *** indicates a *p* value < 0.001).

### Relationships among A2E induced cell senescence, HMGB1 and Caveolin-1

**Figure 5:**
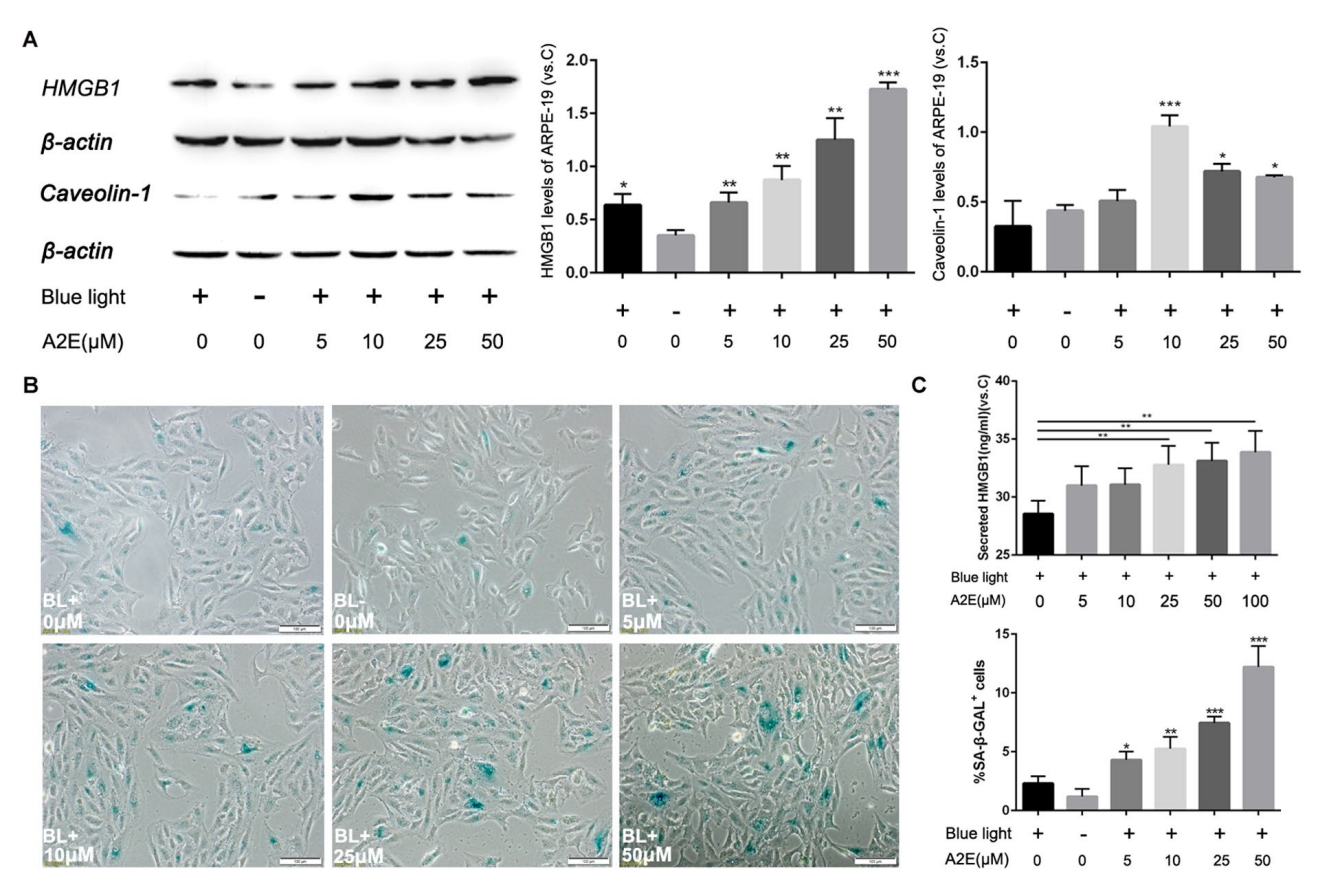
**Blue light exposure of A2E-treated ARPE-19 cells increased HMGB1 and Caveolin-1 expression.** (**A**) Western blot assay for HMGB1 and Caveolin-1 in RPE cells treated with a concentration gradient of A2E with or without blue light, quantified by densitometry, and the results are expressed as a ratio with β-actin. Data are presented as means ± SD; * indicates a *p* value < 0.05, ** indicates a *p* value < 0.01, n=3. (**B**) Representative microscopic images of β-galactosidase staining in RPE cells with various concentrations of A2E. Quantification of percentage of cells with positive SA-β-gal staining.Data are presented as means ± SD; * indicates a *p* value < 0.05, ** indicates a *p* value < 0.01, n=3. (**C**) The release of HMGB1 induced by A2E treatment were detected by ELISA assays.

Because HMGB1 increases expression of Caveolin-1, we further explored the relationship among A2E, HMGB1 and Caveolin-1. We assessed HMGB1 and Caveolin-1 expression in ARPE-19 cells by western blot and found that A2E increased the levels of both compared with unstimulated cells. Interestingly, A2E enhanced expression of Caveolin-1, although Caveolin-1 levels did not increase with higher concentrations of A2E. The tendency of Caveolin-1 expression first increased and then decreased at more than 10 μM A2E (Figure 5A, * indicates a *p* value < 0.05, ** indicates a *p* value < 0.01, *** indicates a *p* value < 0.001). Furthermore, although the tendency of Caveolin-1 expression first increased and then decreased at more than 10 μM A2E, the senescence of cells was still in process (Figure 5B). Since we have found A2E could induce translocation of HMGB1, we collected the supernatants from ARPE-19 cells stimulated by different concentrations of A2E to investigate the level of HMGB1 secretion into the extracellular space. Enzyme Linked Immunosorbent Assay (ELISA) revealed the secretion of HMGB1 was increased along with increasing concentrations of A2E (Figure 5C). These data showed that A2E increases HMGB1 and Caveolin-1 expression, with links to cell senescence, but the expression of Caveolin-1 was changing dynamically based on different A2E concentration.

### Glycyrrhizic acid inhibited the release of HMGB1 alleviated A2E induced cell senescence

**Figure 6:**
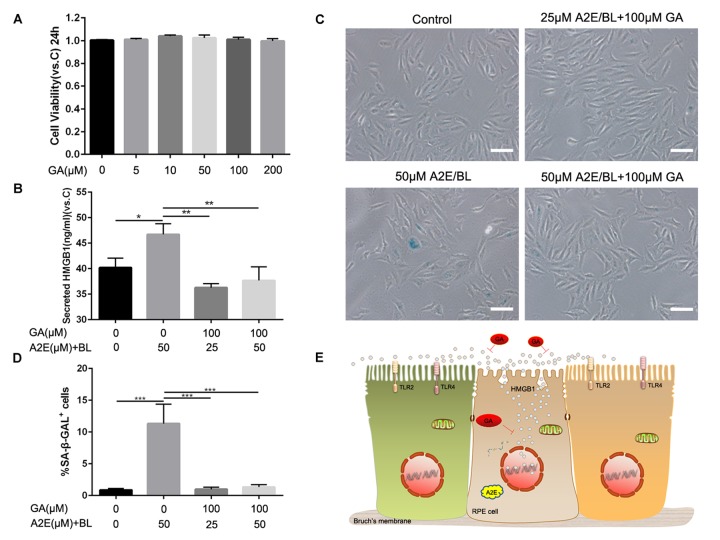
**Glycyrrhizic acid alleviated A2E induced cell senescence.** (**A**) An MTT assay was performed on RPE cells treated with different concentrations of GA. Data are presented as means ± SD; * indicates a *p* value < 0.05, ** indicates a *p* value < 0.01, n=3. (**B**)The release of HMGB1 induced by different concentrations of A2E+BL with or without 100μM GA were detected by ELISA assays. Data are presented as means ± SD; * indicates a *p* value < 0.05, ** indicates a *p* value < 0.01, n=3. (**C**) Representative microscopic images of β-galactosidase staining in RPE cells induced by different concentrations of A2E+BL with or without 100μM GA. (**D**) Quantification of percentage of cells with positive SA-β-gal staining. Data are presented as means ± SD; * indicates a *p* value < 0.05, ** indicates a *p* value < 0.01, n=3. (**E**) Proposed schematic model for strategies for HMGB1 inhibition in response to A2E treatment.

To further confirm the role of ARPE cell-secreted HMGB1 in cell senescence, we used a HMGB1 inhibitor, glycyrrhizic acid (GA) which binds directly to HMGB1, to block HMGB1 released into the extracellular space and inhibit its extracellular cytokine activities [16] (Figure 6E). MTT assay was used to identify candidate concentrations of GA that were not cytotoxic to ARPE-19. Shown in Figure 6A, these data revealed that glycyrrhizic acid showed no toxicity at various concentrations from 5μM to 200 μM. Then we explored the effect of GA on ARPE-19 cells treated with A2E and blue light, and 50 μM A2E induced ARPE-19 cell senescence used as a positive control. The results showed that GA blocked the release of HMGB1 into the extracellular space and A2E induced cell senescence was mitigated correspondingly. (Figure 6B, C, D) These results indicated that blocking HMGB1 by directly inhibiting its extracellular cytokine activities could alleviate A2E induced cell senescence.

## DISCUSSION

Cellular senescence is a process during which physiological function and proliferation and differentiation capacities decline gradually [17]. It is also a state of permanent cellular division arrest that only concerns only mitotic cells. Although RPE cells are quiescent in the retina, they can undergo oxidative stress-induced senescence. Therefore, cellular senescence can be considered as an important molecular pathway of AMD pathology [18]. We chose RPE cells differentiated from iPSCs because their young age allows them to be observed during the process of aging under certain stimulations. This approach has been applied to several studies on age-related diseases, including Parkinson’s disease and Alzheimer’s disease [19,20]. Although it has been reported that A2E accumulation causes RPE cell senescence and dysfunction, including complement factor activation and oxidative stress [21], there is thus far no unanimous conclusion regarding the specific mechanism.

HMGB1 organizes DNA, regulates transcription and is a damage-associated molecular pattern molecule that is related to oxidative stress and downstream apoptosis or survival [22]. Under pathological conditions such as hypoxia, cell death, atherosclerosis and ischaemia-induced angiogenesis [23,24,14] and in senescent cells, HMGB1 is upregulated and translocated from the nucleus to the cytoplasm and extracellular space. Indeed, HMGB1 is deemed a more reliable and accurate evaluation of the senescent state than using SA-β-gal positive staining alone. Importantly, HMGB1 is regarded as a central mediator of senescent phenotypes [25]. After identification by proteomic MS-based measurement, we detected HMGB1 expression and localization in A2E-treated RPE cells and confirmed that the protein was upregulated and released from the nucleus into the cytoplasm. This is consistent with the findings of CoCl_2_-induced hypoxia and senescent human and mouse cells in culture and *in vivo* [25,26]. GA, extracted from the root of *G.glabra* was recently found to suppress HMGB1-induced injury by binding directly HMGB1. Furthermore, the effect of GA was demonstrated against photo-aging in skin, which indicated the potential role of GA against aging [27]. GA can inhibit the release of HMGB1. Alleviated A2E induced cell senescence confirmed the important role of HMGB1 in cell senescence.

Another RPE cell age-related protein detected in our study is Caveolin-1, which is the main component of the caveolae found in most cell types and is involved in the regulation of many cellular processes, such as mitochondrial function, proliferation, migration and senescence [28]. Senescence is strongly associated with decreased responses to growth factors that interact with Caveolin-1 via caveolae [29], and it has been reported that Caveolin-1 plays a major role in both replicative senescence and stress-induced premature senescence [15]. Our results showed that HMGB1 upregulation and release enhanced expression of Caveolin-1, suggesting that both HMGB1 and Caveolin-1 had a synergistic effect on RPE cell senescence. Consistently, Caveolin-1 and translocation of HMGB1 significantly and consistently suppress cancer cell migration and invasion, with little effect on cell viability [30]. HMGB1 binding to RAGE upregulates Caveolin-1 expression during macrophage necroptosis [31]. Therefore, it is worth our studying this synergistic effect in RPE cells.

Although interaction between HMGB1 with Caveolin-1 was indicated by the STRING program, there is no evidence to date for this in RPE cells. Furthermore, in research on the pathophysiology of pre-eclampsia (PE), hypoxic trophoblasts displayed higher intracellular HMGB1 protein levels which could increase TLR4 and Caveolin-1 [14]. However, Shang et al. suggested that RAGE mediated HMGB1-induced Caveolin-1 phosphorylation but did not raise the expression level; Lin et al. showed that Caveolin-1 phosphorylation, which promotes HMGB1 release, regulates endothelial cell apoptosis [32,33]. Therefore, it is also worth addressing how HMGB1 interacts with Caveolin-1.

Upregulation of Caveolin-1 inhibits cell proliferation by suppressing receptor tyrosine kinase activities. In contrast, Caveolin-1 causes an enlarged and flattened shape in senescent cells via upregulation of Rb family and focal adhesion proteins [34]. Furthermore, the senescent phenotype can be reversed by downregulation of Caveolin-1, which suggests that it is a major switch in cellular senescence [35]. Our results demonstrate that cell aging reduces migration and invasion, which was consistent with previous reports [36], and that Zo-1 and β-catenin are upregulated, despite the results of other studies indicating that all tight junction proteins increased [37]. The upregulation of Zo-1 may be associated with activation of Src tyrosine kinases and matrix metalloproteinases (MMPs), which can be negatively regulated by the scaffolding domain of Caveolin-1 [38,39]. For example, Hardyman et al. found that a Src kinase inhibitor was able to rescue structural destruction of the epithelial cell barrier [40], and Vermeer reported that activation of MMP-9 decreased expression of Claudin-1 and Occludin [41]. In addition, upregulating Caveolin-1 rescued expression of tight junction proteins under hyperoxic conditions [37]. Nonetheless, β-catenin is not only a type of tight junction protein but is also a key link for the Wnt pathway, which is related to cell proliferation. Galbiati et al. suggested both Caveolin-1 and β-catenin levels increased and decreased in parallel and Caveolin-1 inhibited Wnt-1 signaling [42]. Kook et al. showed that quercetin could protect RPE cells from oxidative damage and cellular senescence via decreasing the expression of Caveolin-1 [43].

Although our expected results were that expression of Caveolin-1 would increase with increasing concentrations of A2E, it exhibited a tendency of first increasing up to 10 μM A2E and then decreasing above 10 μM A2E. However, there is no relevant research on changes in Caveolin-1 expression during RPE aging. These results may be due to the following reasons. First, we found that cell growth began to decline and that cell morphology began to change under high concentrations of A2E (Figure 5B). Yu et al. confirmed that Caveolin-1 deficiency induces premature senescence, with mitochondrial dysfunction, in human diploid fibroblasts [44]. Second, we surmised that cumulative feedback inhibition of Caveolin-1 may be associated with RPE cell senescence because upregulation of Caveolin-1 inhibits cellular levels of nitric oxide (NO) by regulating NO synthase activity [45]. Regardless, the mechanism responsible for these changes remains to be determined.

In summary, upregulation of HMGB1 and Caveolin-1 caused RPE cell senescence and suppressed migration and invasion, and β-catenin and Zo-1 accumulation was enhanced by A2E in RPE cells. In particular, the results showed a change in expression of HMGB1 and Caveolin-1, which suggests that they are prime gatekeepers in RPE cell senescence. The above results indicate that stabilizing expression of HMGB1 and Caveolin-1 is a potential therapeutic target to prevent the progression of RPE cell senescence.

## MATERIALS AND METHODS

### Cell culture

iPSC-derived RPE cell lines were created from healthy individuals, as described inYang et al. and Lin et al. [6,46]. In short, lentiviral vectors were used to transduce fibroblasts into iPS cell lines with *OCT4, SOC2, KLF4*, and *MYC*, which were cultured in human embryonic stem cell medium with 10 mM basic fibroblast growth factor (FGF).IPS cell lines wereco-cultured with mitomycin-C-treated stromal cells from the PA6 line and were further incubated in differentiation medium under 5% CO_2_ at 37°C. Differentiation medium contained human embryonic stem cell medium (HUESM)–bFGF with 10 nm Nicotinamide (from d 0 to 20) and 20 ng/ml Activin A (from day 20 to 40).The first two generations of cells were plated onto 12-well dishes with feeder cells to induce RPE differentiation. After 6 weeks, pigmented colonies were re-plated on Matrigel-coated plates in RPE culture medium.ARPE-19was purchased from American Type Culture Collection (ATCC, Manassas, VA, USA) and cultured in complete Dulbecco's modified Eagle's medium F-12 nutrient mixture (DMEM F12, Gibco Life Technology, China) containing 10% fetal bovine serum (FBS) and 1% penicillin/streptomycin (100 unit penicillin/100 μg streptomycin per mL) (Invitrogen, USA) at 37°C with 5% CO_2_. All cells were cultured in a humidified 5% CO_2_ atmosphere at 37°C and passaged every 5 to 7 days.

### Proteomic MS-based measurements

The process described in Yang et al. and Lin et al. [6,46] consisted of three steps. (1) The third passage of iPSC-derived RPE cell lines were treated with and without A2E. Three biological replicates were prepared representing three separate cultures derived from each cell line and were also performed separately for A2E-treated samples. (2) Proteins were extracted from each cell line, reduced and alkylated before tryptic digestion, and RapiGest was cleaved with acid. The resulting peptides were analysed using a Synapt G2 quadrupole-time-of-flight mass spectrometer (Waters Corp.) with MSE data-independent scanning. (3) Initial data were processed using ProteinLynx Global Server (Version 2.5 RC9, Waters Corp.). Further analysis was performed with TransOmics software (Waters Corp.) and the NCBI database of human sequences.

### Lentivirus-mediated transduction

For overexpression and knock-down of human HMGB1 and Caveolin-1, lentiviruses were purchased from Genechem (Shanghai, China). For infection of ARPE-19 cells, we used 5μM polybrene, and the medium was changed after 11 h. To acquire stable clones, 2μM puromycin (Solarbio, China) was added to the culture medium, and the medium was replaced every 3 days with fresh puromycin-containing medium until resistant colonies were identified.

### Drug treatment

A2E was purchased from Gene And Cell Technologies (310 Georgia St,Vallejo CA, 94590,USA), dissolved in dimethyl sulfoxide (DMSO) at a concentration of 25 mM and stored at −80°C in the dark as a stock solution. ARPE-19 cells were incubated with different concentrations of A2E in culture medium without FBS for 48 h. After A2E loading, RPE cells were exposed to 470 ± 20 nm light at 2000±500 lx (Yingze, Tianjin) for 10 min, as described previously [47]; the cells were then returned to complete medium and incubated for 24 h. GA was purchased from MCE, ARPE-19 cells were pretreated with GA for 2h, then the cell culture medium was replaced with medium containing A2E and GA for 48h. Mammalian recombinant HMGB1 (rHMGB1) protein was purchased from Sigma–Aldrich, ARPE-19 cells were treated with rHMGB1 for 24h.

### Cell viability assays

Cytotoxicity was assessed using the MTT assay. After treatment with different concentrations of A2E/Blue light, 20 *μ*L MTT labelling reagent (Solarbio, China) was added to 200 *μ*L medium in each well. After 4h of incubation at 37°C, the labelling reagent was replaced with 200 *μ*L DMSO, and the sample was shaken for 15 min to dissolve the crystals. Optical density (OD) was measured at 490 nm using a Full Wavelength Microplate Reader (Infinite 200 PRO, TECAN). FDA/PI staining was also performed.

### Quantitative real-time PCR

Expression of tight junction genes, Claudin-1, Claudin-3, Zo-1, Occludin, N-cadherin, β-catenin, and HMGB1, Caveolin-1, was analysed by RT-PCR. Each gene expression value was normalized to the endogenous control glyceraldehyde-3-phosphate dehydrogenase (GAPDH). RNA was extracted from cells in 6-well plates using 1mL of Trizol® (Invitrogen,USA) and resuspended in 20μL diethyl pyrocarbonate (DEPC)-treated water. The total RNA concentration was determined using a Nanodrop 2000 (Thermo Scientific). Total RNA (1 μg) was used for reverse transcription with a retroscript kit (Revert Aid First Strand cDNA Synthesis Kit, Thermo Scientific), and real-time PCR was performed using a 7900HT Fast Real-Time PCR system (Applied Biosystems, USA). For quantification, the relative expression of different gene transcripts was calculated with the ΔΔCt method. The Ct of any gene of interest was normalized to the Ct of GAPDH. Fold changes were determined as 2 ^− ΔΔCt^. All reactions were performed 3 times. Primer information is provided in Table 2.

**Table 2:** Primers used for quantitative RT-PCR.

	Forward **(5’to 3’)**	Reverse **(5’to 3’)**
GAPDH	TGTGGGCATCAATGGATTTGG	ACACCATGTATTCCGGGTCAAT
HMGB1	GAGAGGCAAAATGTCATCAT	GGGATCCTTGAACTTCTTTT
Caveolin-1	CGGGAACAGGGCAACATCTAC	CTTCTGGTTCCGCAATCACATC
Zo-1	AAGGAAGGCTTAGAGGAAGGTGA	GTCCATAGGGAGATTCCTTTTCA
β-catenin	CCTGAGGAAGAGGATGTGGATAC	CATTTAGCAGTTTTGTCAGTTCAGG
Claudin-1	CTGGGAGGTGCCCTACTTTG	ACACGTAGTCTTTCCCGCTG
Claudin-3	ACGCGAGAAGAAGTACACGG	GTAGTCCTTGCGGTCGTAGC
Occludin	AGGTTCCATCCGAAGCAGG	GTGTAGGTGTGGTGTGTCGG
N-cadherin	CCTTTCAAACACAGCCACGG	TGTTTGGGTCGGTCTGGATG

### Western blot analysis

ARPE-19 cells were collected in RIPA buffer (Solarbio, China) with protease inhibitors (Thermo Fisher Scientific). Protein concentrations were measured using a bicinchoninic acid (BCA) assay kit (Solarbio, China) A total of 20 or 40 μg of protein was loaded per lane, separated by sodium dodecyl-sulfate polyacrylamide gel electrophoresis (SDS-PAGE), and transferred to Immobilon-FL polyvinylidene difluoride (PVDF) membranes. Subsequently, the membranes were blocked in 10% bovine serum albumin (BSA, BD Biosciences) in phosphate-buffered saline with Tween 20 (PBST) for 1.5 h at room temperature and incubated overnight at 4°C with primary antibodies against TLR2(1:1000, Rabbit, Abcam), TLR4(1:2000, Mouse, Proteintech), RAGE(1:1000, Rabbit, Abcam), Tublin(1:1000, Mouse, Proteintech), β-actin (1:1000, Mouse, Abcam), Caveolin-1 (1:10000, Rabbit, Abcam), HMGB1 (1:1000, Mouse, Abcam), Zo-1 (1:1000, Rabbit, Proteintech), or β-catenin (1:5000, Rabbit, Abcam). After washing 3 times, the membranes were incubated for 2 h in the dark at room temperature with horseradish peroxidase (HRP)-conjugated secondary antibodies diluted 1:3000 in PBST. The membranes were washed in PBST 3 times before visualizing using Immobilon Western Chemiluminescent HRP Substrate (MILLIPORE, USA). Blots shown are representative of at least three biological repeats for each experiment. Every immunoreactive band was detected using the ECL detection system (UVP, USA), and densitometric values were quantitated using ImageJ software (version 1.45). The relative expression of each immunoreactive band was normalized to that of β-actin.

### Immunofluorescence and confocal microscopy

ARPE-19 cells in a 24-well cell culture plate were fixed with 4% paraformaldehyde (PFA) for 20 min and then permeabilized in 0.1% Triton X-100 in PBS for 20 min at room temperature. After being blocked with10% goat serum in PBS for 1.5 h, the samples were incubated with the primary antibodies described above at 4°C overnight. Coverslips were washed with PBST 3 times and incubated with Alexa Fluor 488-conjugated (1:500; ThermoFisher) and Alexa Fluor 594-conjugated (1:500; ThermoFisher) secondary antibodies diluted in PBST at room temperature in the dark for 1 h. Coverslips were washed 3 times and stained with 4′,6-diamidino-2-phenylindole (DAPI) for 3-5 min and then imaged by confocal microscopy (Zeiss).

### Migration assays

For the wound-healing assay, cells were transfected with Caveolin-1 for 24 h and then cultured in 6-well plates. When reaching 80% confluence, we used a sterilized pipette to scratch the cell monolayer. After washing three times with PBS, the cells were cultured in medium without FBS. Images shown are from three time points, 0, 12 and 24h, and demonstrate wound closure, which was measured using ImageJ. For transwell assays, cells were transfected with Caveolin-1 for 24 h, trypsinized and counted; 1 × 10^5^ cells in medium without FBS were placed in the top chamber of a Transwell device (24-well insert; 8 μm, pore size; Corning Incorporated). Medium with 20% FBS was used as a chemical attractant in the lower chamber. After incubation at 37°C for 24 h, the membranes were fixed with 4% PFA for 30 min and stained. Cells migrated to the lower side of the membranes were counted using an inverted microscope.

### ELISA assays

The amount of HMGB1 in cell culture medium was assessed using the HMGB1 ELISA kit(Mlbio, China).

### SA-β-gal staining

The SA-β-gal staining assay was performed using an SA-β-gal staining kit (Solarbio, China) following the manufacturer’s instructions.

### Data and statistical analysis

All figures are representative of at least 3 separate experiments. All quantitative data were analysed with SPSS (Version 22), and the results are expressed as the mean ± SEM, with *p*< 0.05 considered statistically significant. Differences between groups were assessed using either an independent *t*-test or one-way analysis of variance (ANOVA) with Tukey's post hoc or Dunnett's tests.
